# Creasote and Its Elements

**Published:** 1892-10

**Authors:** 


### Creasote and Its Elements.

Dr. E. Main (*Bulletin Generale de Therapeutique*, 1892, liv. 10e, p. 205) has made a laboratory study of this remedy, which has of late attracted so much attention. He established the fact that the elements of creasote were poisonous in the following order: (1) para-cresylol (least); (2) phlorol; (3) guaiacol; (4) creasote; (5) creasol (most). Locally, creasol was the most irritant, guaiacol the least. For all these elements this laboratory work shows three important characteristics; (1) that they are feebly poisonous; (2) a tolerance can be established; (3) they are eliminated by the lungs. As remedies against tuberculosis they can be arranged in the following order: (1 and 2) phlorol and creasol; (3) para-cresylol; (4) guaiacol; (5) creasote (most powerful).

It is believed that although all the elements of creasote have some value, and indeed guaiacol should be especially mentioned, yet creasote is the most active. Beechwood cresaote should be preferred for its antiseptic power, for its feeble toxicity, and because of the results furnished by experimental therapeutics as well as by clinical observation.—*Med. and Surg. Reporter.*

